# Making students' marks fair: standard setting, assessment items and post hoc item analysis

**DOI:** 10.5116/ijme.54e8.86df

**Published:** 2015-02-28

**Authors:** Mohsen Tavakol, Gillian A. Doody

**Affiliations:** 1 Assessment Unit, School of Medicine, The University of Nottingham, UK

**Keywords:** standard setting, assessment items, post hoc item analysis

Establishing test marks and reporting them to students is a difficult task for key medical educators. Failing a test is usually an unpleasant experience for any student. Therefore, medical educators must ensure the "reasonableness" of the pass mark and additionally, the quality of all test items. A poorly established pass mark and/or defective items may increase the number of students who fail unfairly. Furthermore, student complaints, legal actions and political issues may arise when pass marks are determined with poor procedural, internal or external validity. In any criterion-referenced assessment, the process of post hoc item judgment is essential and should be considered as a vital step in the validation of the standard setting method.

## Benefits of using post hoc item data

Standard setting should aim to accurately pinpoint any individual student's performance along a continuum. Therefore, pass mark setters should be aware of the ability range of their students, the upper and lower limits of the continuum, when undertaking the task of rating items during standard setting. By reference to post hoc impact data (i.e. students actual performance data), standard setters may gain greater understanding of the performance of their students when judging future items. Post hoc item analysis can also encourage discussion between standard setters and thereby helps to minimise subjective errors of standard setters when judging student ability in future.

It is important to remember, that when standard setters establish a reasonable pass mark, those students who have received a mark immediately on either side of the pass mark are actually very similar in terms of their performance.^1^ Furthermore, the reliability of the initial standard setting process also affects the accuracy of these borderline Students' marks. Therefore, an argument for a post hoc evaluation of test items to ensure that items perform as predicted during standard setting, easily develops. The most common standard setting discrepancies, seen after a test has been performed, are produced by aberrant items. Flawed assessment items may affect the classification of students i.e. pass or fail. 'If there are defective items in a test, the students should not be held accountable for them'.^2^ Students' raw marks are not true marks (free of error) and therefore may not accurately reflect student ability. Therefore, final marks should not be reported until after the post hoc analysis of individual item performance. Student marks should be moderated, before final results are announced.

## Plausible methods to perform post hoc moderation

In medical education assessments, candidates are measured in various situations by various assessors, against predetermined standards, to measure performance e.g. assessor and standardised patient ratings of students in objective structured clinical examinations (OSCEs), ratings of knowledge questions in standard setting, marking of written assignments and presentations, or judgements relating to attitude and behaviour in respect to professionalism. Systematic errors (hawks and doves) and random errors (made by unreliable or inconsistent raters) can have a detrimental effect on student marks.

By detecting and adjusting unreliable ratings, student marks become a more robust representation of actual performance, as the reliability of ratings is significantly increased.^3^^, ^^4^ To correct the effects of systematic errors, Item Response Theory (IRT) models, Ordinary Least Squares or Weighted Least Squares may be applied.^5^^, ^^6^ The IRT analysis using a Multifaceted Rasch model or Generalisability studies provide an estimate of the effect of differences in the assigned ratings by the raters.

With respect to a standard setting practice, IRT models can provide useful information for standard setters by comparing the consistency of their ratings with IRT model estimates. If there is a discrepancy between standard setters' ratings and the IRT estimate, a new pass mark may be established by marking all students as having correctly responded to aberrant items. Individual item analysis can therefore contribute significantly to the moderation of student pass marks.

Using the IRT models, standard setters are able to identify any items that are not mapped to student ability i.e. items which are either too difficult or too easy for that cohort. By adjusting for these items, medical educators can convert Students' raw marks to moderated final marks. For example, if an item is too difficult for the cohort, with only 10% getting the correct answer that mark is given to all the cohort, including the 90% who answered incorrectly. It would be erroneous to simply remove the question as this would discriminate negatively against the 10% of students who answered correctly initially.

## Standard Error of Measurement to perform post hoc item moderation

Estimating the standard error of measurement (SEM) provides valuable information about the errors attached to 'raw' student marks. Defective items can increase the SEM in a test, so such items can increase the number of failures unfairly as 'in most medical examinations, which are pass/fail, the only candidates who will be affected by error within the exam are those around the pass mark'.^7^ By calculating the absolute error variance of a test using Generalisability theory,^8^ its square root equals the absolute SEM, we are now in a position to create a range pass mark for a test without changing the standard setting method. This is especially important if sequential testing is being used to determine which students require further testing to demonstrate competency.

Taken together, students are sensitive to their marks, and they can sense whether or not their marks are fair. Undertaking a moderation process, prior to reporting final marks, will make student marks fair, and increase student satisfaction with the process of the exam cycle.
